# Plant Identification Based on Leaf Midrib Cross-Section Images Using Fractal Descriptors

**DOI:** 10.1371/journal.pone.0130014

**Published:** 2015-06-19

**Authors:** Núbia Rosa da Silva, João Batista Florindo, María Cecilia Gómez, Davi Rodrigo Rossatto, Rosana Marta Kolb, Odemir Martinez Bruno

**Affiliations:** 1 São Carlos Institute of Physics, University of São Paulo, PO Box 369, 13560-970, São Carlos, SP, Brazil; 2 Institute of Mathematics and Computer Science, University of São Paulo, USP, São Carlos, São Paulo, Brazil; 3 Department of Physics, Faculty of Biochemistry and Biological Sciences, National University of Littoral, Santa Fe, Argentina; 4 Department of Applied Biology, Faculty of Agriculture and Veterinary Sciences, Univ Estadual Paulista, UNESP, Jaboticabal, São Paulo, Brazil; 5 Department of Biological Sciences, Faculty of Sciences and Letters, Univ Estadual Paulista, UNESP, Assis, São Paulo, Brazil; Medical University of Graz, AUSTRIA

## Abstract

The correct identification of plants is a common necessity not only to researchers but also to the lay public. Recently, computational methods have been employed to facilitate this task, however, there are few studies front of the wide diversity of plants occurring in the world. This study proposes to analyse images obtained from cross-sections of leaf midrib using fractal descriptors. These descriptors are obtained from the fractal dimension of the object computed at a range of scales. In this way, they provide rich information regarding the spatial distribution of the analysed structure and, as a consequence, they measure the multiscale morphology of the object of interest. In Biology, such morphology is of great importance because it is related to evolutionary aspects and is successfully employed to characterize and discriminate among different biological structures. Here, the fractal descriptors are used to identify the species of plants based on the image of their leaves. A large number of samples are examined, being 606 leaf samples of 50 species from Brazilian flora. The results are compared to other imaging methods in the literature and demonstrate that fractal descriptors are precise and reliable in the taxonomic process of plant species identification.

## Introduction

A series of methodologies and approaches have been performed in the task of understanding and description of the natural world surrounding us [1]. All major areas of scientific knowledge, as geology, physics, biology and medical sciences have been searching for patterns that may help in the understanding of natural phenomena [1–3]. In Biology, such aspects started in the ancient Greece, where philosophers tried to describe, identify and classify natural entities (species) based on identifiable traits [4]. The Greek philosopher Theoprasthus performed the most famous case, where he proposed a classification system of plant species according to their external morphology, adopting as a classifier their distinct growth forms [5]. Since these ancient times, scientists have proposed a series of manners to perform classification [6–8] and to identify species. Yet, in plants, the older and most adopted methodology used to infer and produce classification system is the observation and description of internal and external plant traits [9, 10], associated, in recent times, with the information stored at molecular level [11].

The most common aspects used by specialists to categorize and identify species concern the use of external traits of plants, in where such specialists access information stored in the form, ontogeny and number of elements forming reproductive organs (flowers) and dispersion entities (fruits) [12, 13]. The use of such elements produced both good tools to identify species and important classification systems to understand the evolution of groups of species [8, 14]. Despite the importance and significance of such aspects, the analysis of such structures cannot be always employed, as these elements appear only in specific times of year, when plants are reproducing or dispersing their descendants [15]. In such cases, specialists also recur and extract information stored in vegetative parts of plants, especially the leaves, which are available for sampling throughout the year [16].

When assessing vegetative organs as the leaves, there is a chance to confound certain information provided by their morphological and anatomical analysis [17, 18], as leaves are one of the most diverse plant organs in terms of morphology and anatomy [19, 20] and such morpho-anatomical traits can vary drastically according to environmental conditions [21]. However, some studies have provided good evidence that the analysis of certain external and internal leaf structures could be of substantial information to aid species classification [22–24]. Until recently, information stored on vegetative traits of plants were only extracted by the human eye, which is capable of extracting low amounts of information such as shape, types, divisions, among others. Nowadays, a series of computational methodologies are available to search and extract information to discriminate plant species [21, 25], assessing properties such as texture and color, which were not possible to be inferred by conventional analysis. The use of such approaches has been explored with great success, using both external [26–29] and some internal [25] aspects of leaves.

Among the computational analysis of leaf internal structures, only color and texture information of photosynthetic and protection tissues have been explored with success to discriminate plant species [25]. Nevertheless, leaves have a great diversity of other internal structures that can potentially store information for discrimination patterns [13, 30]. One of them is the midrib, which drastically differs between species in its shape and composition of vascular and fundamental tissues [20]. Anatomically, leaf midrib is composed by a set of highly specialized tissues (pholem and xylem) and other cells, which are normally very similar between individuals of the same species [31], as this region is less plastic than other regions of the leaf blade, as the mesophyll for example [32]. Additionally, the midrib is considered as a stable region regarding the conservation of its structures when submitted to the image acquisition process. The use of midrib anatomy to discriminate plant species has been recently explored as a new tool to assist plant classification [33, 34]. Such studies indicate the great potential of the computational methodologies to explore the patterns of composition and arrangement of tissues and structures in the midrib, which may provide a great additional source of information to the discrimination of plant species. In fact, a preliminary approach using only 10 species provided evidence for the robustness of such kind of methodology [35].

Considering the several methodologies used to discriminate plant species, many of them successfully made use of latest and advanced methods of image analysis. Most of such methods analyze only the external shape of the leaf; although this can be sufficient in some situations, the addition of internal traits, such as that from midrib, may provide the creation of robust descriptors, able to synthesize all this informational richness in a feature vector, making the discrimination of plants a more feasible task. The efficiency of this kind of analysis turns state-of-the-art texture-based methods, like LBP and Gabor-wavelets, into potentially good methods for the automatic identification of species studied here. For instance, Casanova et al. [36] obtained good results by extracting texture features from the leaf surface using Gabor wavelet filters. Still among the texture-based imaging methods in plant leaves, fractal descriptors have demonstrated to be a promising approach mainly to identify species based on the digital representation of the leaf [26, 37, 38]. This is a consequence of the complex nature intrinsic to fractals, which makes them quite similar to much structures found in the nature and, particularly, in the plant leaves.

Based on the context exposed above, in our study we have applied a combination between two advanced computational methods (fractal-based descriptors, that is, Bouligand-Minkowski [38] and Fourier [39]) to extract and provide species discrimination based on information stored in leaf midribs. The results obtained using 606 leaf samples of 50 species from Brazilian flora demonstrated the robustness of applying this methodology.

## Materials and Methods

### Image Acquisition

Samples of leaves were collected from 50 species in the Cerrado biome in central Brazil, at IBGE Ecological Reserve (Table 1). IBGE (Brazilian Institute of Geography and Statistics) allows the use of samples for scientific research purposes. At least four leaves (one per individual) were sampled for each species. All samples were obtained from fully expanded leaves collected from the third and fourth nodes from the branch tip. Middle regions of the leaf, including the midrib, were fixed in FAA 70 (Formalin, Acetic acid, 70% Alcohol) for 48 hours [40]. These were dehydrated in an ethanol series and embedded in paraffin. The thickness of the cross sections was 8*μ*m. The sections were stained with astra blue 1% and basic fuchsin 1%, both from Sigma, and mounted with Entellan®. The images of midribs were captured in 10x objective lens, using a trinocular microscope Axio Lab A1 coupled to a digital camera Axiocam ICc 1.

**Table 1:** Family, species and number of samples (*n*) per species used in the experiments.

Family	Species	*n*
Anacardiaceae	*Anacardium humile* A. St.-Hil.	11
	*Myracrodruon urundeuva* Allemão	14
	*Tapirira guianensis* Aubl.	11
Annonaceae	*Annona crassiflora* Mart.	12
	*Cardiopetalum calophyllum* Schltdl.	11
	*Duguetia furfuracea* (A.St.-Hill.) Saff.	10
Apocynaceae	*Aspidosperma subincanum* Mart.	12
Araliaceae	*Schefflera macrocarpa* (Cham. & Schltdl.) Frodin	11
Aristolochiaceae	*Aristolochia galeata* Mart. & Zucc.	12
Asteraceae	*Baccharis salzmannii* DC.	12
	*Eremanthus glomerulatus* Less.	12
	*Lepidaploa aurea* (Mart. ex DC.) H.Rob.	11
Bignoniaceae	*Arrabidaea brachypoda* Bur	10
	*Jacaranda ulei* Bureau & K. Schum.	20
	*Handroanthus impetiginosus* (Mart. ex DC.) Mattos	10
	*Zeyheria montana* Mart.	11
Calophyllaceae	*Calophyllum brasiliense* Cambess.	12
	*Kielmeyera abdita* Saddi	12
Combretaceae	*Combretum duarteanum* Cambess.	11
Dilleniaceae	*Davilla elliptica* A. St.-Hil.	12
Euphorbiaceae	*Cnidoscolus vitifolius* (Mill.) Pohl	11
	*Maprounea brasiliensis* A.St.-Hill.	11
	*Maprounea guianensis* A.St.-Hil.	12
Fabaceae	*Bauhinia pulchella* Benth.	20
	*Bauhinia ungulata* L.	20
	*Piptadenia gonoacantha* (Mart.) J.F.Macbr.	20
Malpighiaceae	*Byrsonima laxiflora* Griseb.	12
	*Byrsonima subterranea* Brade & Markgr.	11
	*Byrsonima verbascifolia* (L.) Rich. ex A. L. Juss.	12
	*Banisteriopsis stellaris* (Griseb.) B.Gates	15
Malvaceae	*Cavanillesia arborea* (Willd.) K.Schum.	12
	*Eriotheca pubescens* (Mart. & Zucc.) Schott & Endl.	12
	*Guazuma ulmifolia* Lam.	11
	*Sterculia striata* A.St.-Hil. & Naud.	10
Melastomataceae	*Ossaea congestiflora* (Naudin) Cogn.	12
Nyctaginaceae	*Guapira areolata* (Heimer) Lundell	10
	*Guapira noxia* (Netto) Lundell	10
Passifloraceae	*Passiflora clathrata* Mast.	11
Primulaceae	*Myrsine ferruginea* R.B. ex Roem. & Schult	11
Rubiaceae	*Cordiera macrophylla* (K. Schum.) Kuntze	13
	*Sabicea brasiliensis* Wernham	11
	*Tocoyena formosa* (Cham. & Schltdl.) K.Schum.	11
Rutaceae	*Esenbeckia pumila* Pohl.	11
Sapindaceae	*Cupania vernalis* Cambess.	11
	*Dilodendron bipinnatum* Radlk.	11
	*Serjania lethalis* A.St.-Hill.	12
Smilacaceae	*Smilax campestris* Griseb.	12
Solanaceae	*Solanum lycocarpum* A.St.-Hill.	11
Symplocaceae	*Symplocos mosenii* Brand.	11
Vitaceae	*Cissus erosa* Rich.	12

**Fig 1:**
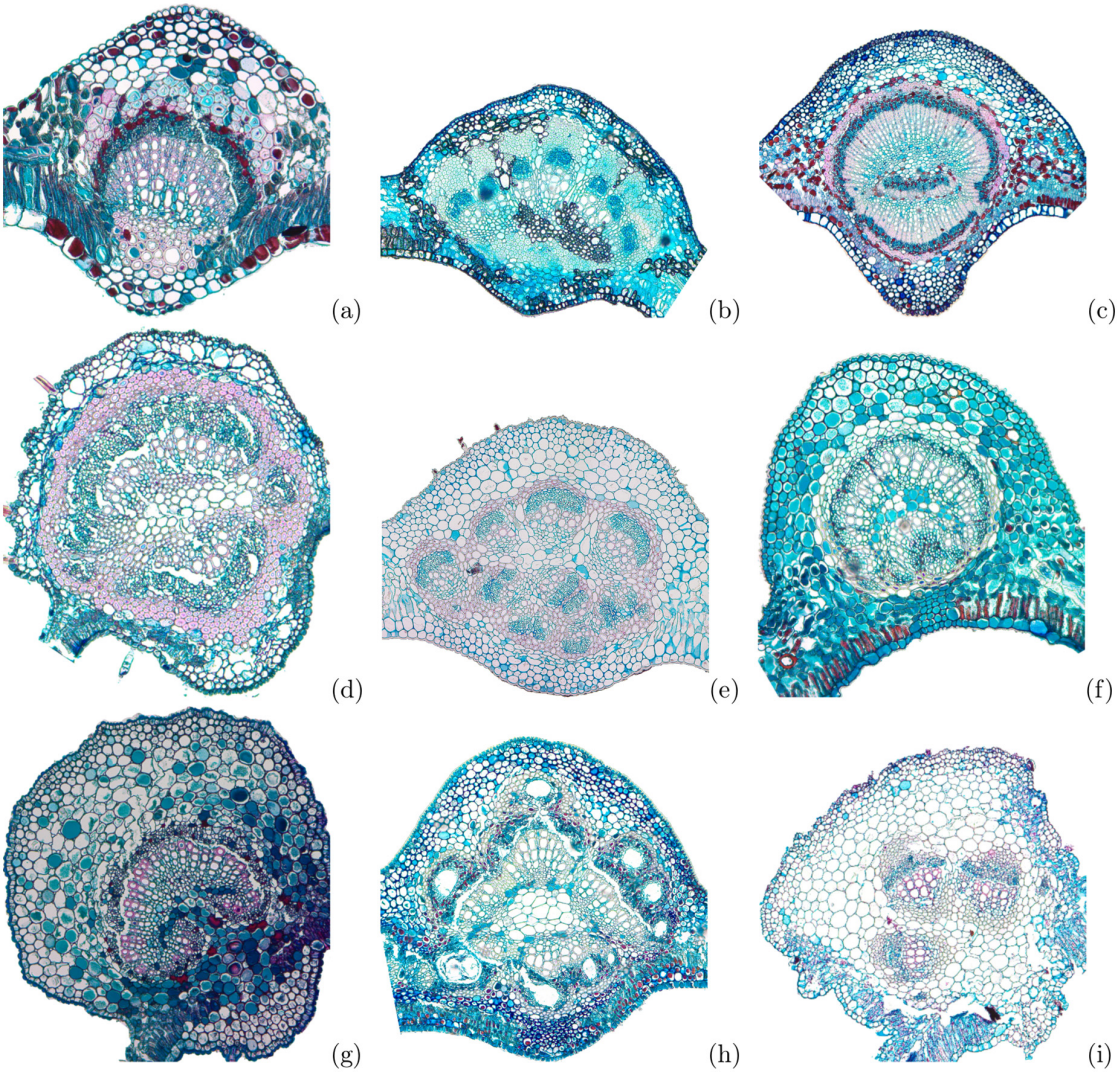
Histological samples of some leaves midrib cross-section used in the experiments. (a) *Banisteriopsis stellaris*, (b) *Cardiopetalum calophyllum*, (c) *Cordiera macrophylla*, (d) *Dilodendron bipinnatum*, (e) *Guapira noxia*, (f) *Myrsine ferruginea*, (g) S*abicea brasiliensis*, (h) *Tapirira guianensis* and (i) *Lepidaploa aurea*.

The image was pre-processed to remove the background by manually segmenting the region of interest, so that only the region of the midrib was analyzed by the fractal descriptors, as shown in Fig 1. In the following, the combination of Bouligand-Minkowski/Fourier fractal descriptors proposed in this study was used to obtain the meaningful features of each sample. Finally, these features are employed in the input of a supervised classifier, which predicts the species of each sample. The classification scheme divides the samples into a training and a testing set, using a 10-fold cross-validation procedure, as described in [41]. The classifier was the Linear Discriminant Analysis (LDA) [41], which has demonstrated to be a suitable method for plant image analysis [38]. The results were compared to other state-of-the-art and classical descriptors, that is, Local Binary Patterns [42] and Gabor-wavelets Descriptors [43].

### Fractal Geometry

A fractal is a geometric structure characterized by two main properties: infinite self-similarity, that is, at any scale, the object is composed by copies of itself, and infinite complexity, that is, there are different details to be observed at any scale.

The most important measure of a fractal is its fractal dimension. This measures how the structure changes (in terms of self-similar patterns) according to the scale. In this sense, it also measures the spatial occupation of the object. Given a geometrical object *X*, one can always measure its length *N* using a rule with length *u*. Although intuitively the length should scale linearly with *u*, in fractals this relation is exponential and the fractal dimension *D*_*X*_ of *X* is given by:

$$\begin{array}{c} D_{X}\propto \lim_{u\to 0}\frac{\log N}{\log u}. \end{array}$$

In the real-world there is no fractal structure, in the strict sense of the word, even because the range of scales is always finite. However, it is quite common to find objects with high complexity and self-similarity at particular ranges of scales. Based on such observation, several methods have been proposed to obtain meaningful information about an object based on a fractal geometry modeling [44–46]. Most of these studies employ the fractal dimension, alone or associated to other traditional measures. There are a number of methods to estimate the fractal dimension *D*_*R*_ of real-world objects. Each one may result in a different value and is more useful for a particular application, but all of them are based in the following bilogarithmic expression:

$$\begin{array}{c} D_{R}\propto \lim_{\epsilon \to 0}\frac{𝔐(\epsilon )}{\log\epsilon ,} \end{array}$$

where 𝔐 is the fractality measure and is specific for each method and *ϵ* is the scale parameter.

Even though the fractal dimension is a powerful descriptor and enough to model some complex systems, it has some outstanding drawbacks. First, it is a unique real value and cannot express all the richness of a structure at all scales. Besides, unlike the case of mathematical fractals, the fractal dimension of real-world objects changes depending on the scale range considered. To make possible a more robust analysis based on fractal geometry, some methods that extend the fractal dimension concept have been proposed, such as the multifractals [47, 48], the multiscale fractal dimension [26, 49] and the fractal descriptors [50, 51]. This study focus on fractal descriptors, given the remarkable results achieved by this approach in previous studies on plant image analysis [26, 29, 37, 38].

#### Fractal Descriptors

Fractal descriptors [37, 50, 51] extend the fractal dimension concept by using all the values in the fractality function. In this way, the set of features (descriptors) *d* are given by:

$$\begin{array}{c} 𝔇:\log\epsilon \to \log 𝔐(\epsilon ). \end{array}$$

The values of this function can be used directly [38] or after a transform to highlight some particular characteristic of the features [51]. They also can be extracted from the entire image [50] or using a recursive decomposition [51]. In any case, they quantify the morphology of the object of interest and its spatial distribution.

## Proposed Methodology

The structural morphology quantified by fractal descriptors is of great importance in the analysis of any natural structure and particularly to describe the shape and visual textures of plant leaves, since the leaf morphology is directly affected by its biological structure and evolutionary history. These are key elements to determine the species to which each sample belongs. A number of studies proposed in the literature confirms the efficiency of fractal descriptors in the analysis of leaves. For example, in [37] and [26], fractal descriptors were employed to identify plant species based on the leaf shape with a good accuracy, whereas in [38] the visual texture of the leaf was quantified by means of fractal descriptors and the results confirmed the precision of fractal descriptors as well.

Here, we propose to employ fractal descriptors to identify species from a tropical savanna of Brazil called “Cerrado” using microscope images from cross-sections of the leaf. Better than scanned or photographed images of entire leaves, the histological sections are capable of providing details of biological structures of the plant. The histological images are pre-processed with the aim of segmenting the midrib removing the background and then they are analyzed both in terms of their shapes and of their visual texture. Then, two different approaches of fractal descriptors, that is, Bouligand-Minkowski [38] and Fourier [39], are extracted from the images and all the descriptors are combined using a Karhunen-Loève representation [41]. These steps are better detailed in the next sections.

### Bouligand-Minkowski Fractal Descriptors

Proposed in [37], the Bouligand-Minkowski fractal descriptors of a gray-level image are obtained from the values of dilation volumes used to compute the Bouligand-Minkowski fractal dimension [38]. These descriptors have demonstrated to be a powerful method to analyze plant structures [38].

Let *I*:[1:*M*] × [1:*N*] → ℜ be a function representing the gray-level image. The first step is to map such image onto a three-dimensional surface *S*, where each pixel in the coordinate (*x*, *y*) is mapped onto a point with coordinates (*x*, *y*, *I*(*x*, *y*)):

$$\begin{array}{c} S=\{(x,y,z)|(x,y)\in [1:M]\times [1:N],z=I(x,y)\}. \end{array}$$

In the following, the surface is dilated by a sphere with radius *r*, that is, each point with coordinates (*x*, *y*, *z*) is replaced by a sphere with center at (*x*, *y*, *z*) and radius *r* and the dilated structure corresponds to the points pertaining to the union of such spheres. The radius is increased up to a pre-defined maximum *r*_*max*_ and the volume of the dilated surface *V*(*r*) is given by:

$$\begin{array}{c} V\left(r\right)=\sum \chi_{𝔇(r)}\left[\left(x,y,z\right)\right], \end{array}$$

where *χ* is the indicator function and 𝔖(*r*) is the set of points in the dilated structure:

$$\begin{array}{c} 𝔖\left(r\right)=\{\left(x,y,z\right)|{\left[{\left(x-P_{x}\right)}^{2}+{\left(y-P_{y}\right)}^{2}+{\left(z-P_{z}\right)}^{2}\right]}^{1/2}\leq r\}, \end{array}$$

where (*P*_*x*_, *P*_*y*_, *P*_*z*_) ∈ *S*.

The Bouligand-Minkowski descriptors 𝔇_*BM*_ are obtained by

$$\begin{array}{c} {𝔇}_{BM}{=\log V\left(r\right)|}_{r=0}^{r_{max}}. \end{array}$$

### Fourier Fractal Descriptors

**Fig 2:**
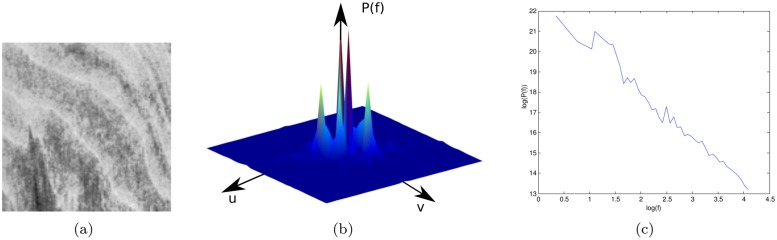
Fourier method to estimate the fractal dimension. (a) A texture image. (b) Fourier spectrum *P*(*f*). (c) Plot of log(*P*(*f*)) × log(*f*). This curve provides the descriptors of the texture.

Fourier fractal descriptors [39] are named after the Fourier fractal dimension. This is computed from the logarithmic relation between the Fourier power spectrum and the frequency (Fig 2). At first, the Fourier transform ℑ of the image is obtained by:

$$\begin{array}{c} ℑ\left(u,v\right)=\int_{-\infty }^{+\infty }\int_{-\infty }^{+\infty }I\left(x,y\right)e^{j2\pi (ux+vy)}dxdy, \end{array}$$ (1)

where *j* is the imaginary number and *u* and *v* are the orthogonal components of the frequency $f=\sqrt{u^{2}+v^{2}}$. The resulting data is composed by complex numbers without any physical meaning, suggesting to use other measures obtained from the transform, like the power spectrum *P*, given by:

$$\begin{array}{c} P=R^{2}+J^{2}, \end{array}$$ (2)

where *R* and *J* are, respectively, the real and imaginary parts of the transform. As stated in [39], the following empirical law is observed for any fractal-like structure:

$$\begin{array}{c} \log\left(P\right)\propto \log{\left(f\right)}^{\alpha }, \end{array}$$

where *α* is a non-negative real-valued exponent used to estimate the fractal dimension.

The Fourier fractal descriptors, within an empirically determined range of frequencies [*f*_*min*_, *f*_*max*_], are given by

$$\begin{array}{c} {𝔇}_{F}{=\log P\left(f\right)|}_{f_{min}}^{f_{max}}. \end{array}$$

### Karhunen-Loève Transform

Let the Bouligand-Minkowski descriptors be represented by a vector with *n*_1_ components, $\vec{{𝔇}_{BM}}=\{x_{1},x_{2},...,x_{n_{1}}\}$, and the Fourier descriptors by a vector with *n*_2_ components, $\vec{{𝔇}_{F}}=\{y_{1},y_{2},...,y_{n_{2}}\}$. The feature matrix of a database of *m* texture images contains in each row the descriptors of each image. For the above descriptors, we have $M_{m\times n_{1}}^{\left(1\right)}$ for the Bouligand-Minkowski descriptors and $M_{m\times n_{2}}^{\left(2\right)}$ for the Fourier descriptors.

For each feature matrix, covariance matrix Σ is provided by:

$$\begin{array}{c} \Sigma \left(i,j\right)=\frac{\sum_{i=1}^{n}\left(M\left(.,i\right)-\bar{M(.,i)}\right)\left(M\left(.,j\right)-\bar{M(.,j)}\right)}{n-1}, \end{array}$$ (3)

where *n* is the number of columns in the feature matrix, *M*(., *i*) represents the column *i* of *M* and $\bar{M(.,i)}$ is the average column-vector.

The next step is to compute the eigenvalues and eigenvectors of Σ. A non-null vector **e** is an eigenvector of Σ if:

$$\begin{array}{c} \Sigma e=\lambda e, \end{array}$$ (4)

for any real value *λ*. *λ* is an eigenvalue of the matrix.

The eigenvalues of Σ are sorted decreasingly *λ*_1_ ≥ *λ*_2_ ≥ …*λ*_*n*_ and the respective eigen-vectors **e_1_**, **e_2_**, …, **e_n_** are the columns of a linear transform matrix *U*.

The descriptor matrices *M* ^(1)^ and *M* ^(2)^ are horizontally concatenated into *M* ^(*C*)^, such that each row of *M* ^(*C*)^ is given by *x*_1_, *x*_2_, *x*_*n*_1__, *y*_1_, *y*_2_, …, *y*_*n*_2__. In the following, the combined matrix is multiplied by the transpose of *U* giving rise to the transformed matrix:

$$\begin{array}{c} D^{\left(C\right)}=U^{T}M^{\left(C\right)}. \end{array}$$ (5)

Finally, the row-vectors of *D* ^(*C*)^ are the fractal descriptors used in this study for the analysis of the leaves. The combination of a spatial and a frequency fractal approach allows for rich and precise descriptors, as they give information concerning the spatial distribution of the midrib as well as how the energy scales with each frequency in the image representation and giving a signature of the distribution of details in multiple scales.

## Results and Discussion

Table 2 shows the performance of different texture descriptors in the identification of the analyzed plant species. Besides the ratio of samples correctly classified (Success Rate) and the respective cross-validation error, the table also shows three other statistical metrics regarding the robustness of the result, i.e, *κ*-index, success reliability (SR) and error reliability (ER). The *κ*-index quantifies (in statistical terms) how better the classifier is than a random classification. Reliability refers to consistency, it measures the degree of reality and stability of a measurement, evaluating if the measure will be the same in every execution. Success and error reliability are metrics derived from the *a posteriori* probabilities of the classifier, being the average *a posteriori* probability for samples correctly and incorrectly classified, respectively. For each sample, classifiers like LDA output one probability score for each possible class and the class assigned to the sample is that having the highest probability. A reliable method is expected to have this highest probability significantly larger than the sum of all the other probabilities and this is what is assessed by the reliability metric. Generally speaking, the proposed method achieved the greatest rate of plants classified correctly, with a substantial advantage over other classical and state-of-the-art approaches, like LBP for instance. It also presented the highest *κ* index and a more robust reliability (Gabor presented the same SR, but much smaller ER, while LBP presented smaller values for both SR and ER).

**Table 2:** Success rates and other statistical measures of the proposed method compared to other literature approaches to classify the same set of plant species.

Method	Success Rate (error) (%)	*κ*-index	SR	ER
LBP	49.12(± 0.6)	0.4641	0.4511	0.6182
Gabor descriptors	42.04(± 0.7)	0.4062	1.0000	0.2809
Fourier fractal descriptors	45.31(± 0.5)	0.4500	0.9651	0.6979
Bouligand-Minkowski fractal descriptors	72.81(± 0.5)	0.7189	1.0000	0.9541
Combined fractal descriptors	83.67(± 0.7)	0.8248	1.0000	0.9693

**Fig 3:**
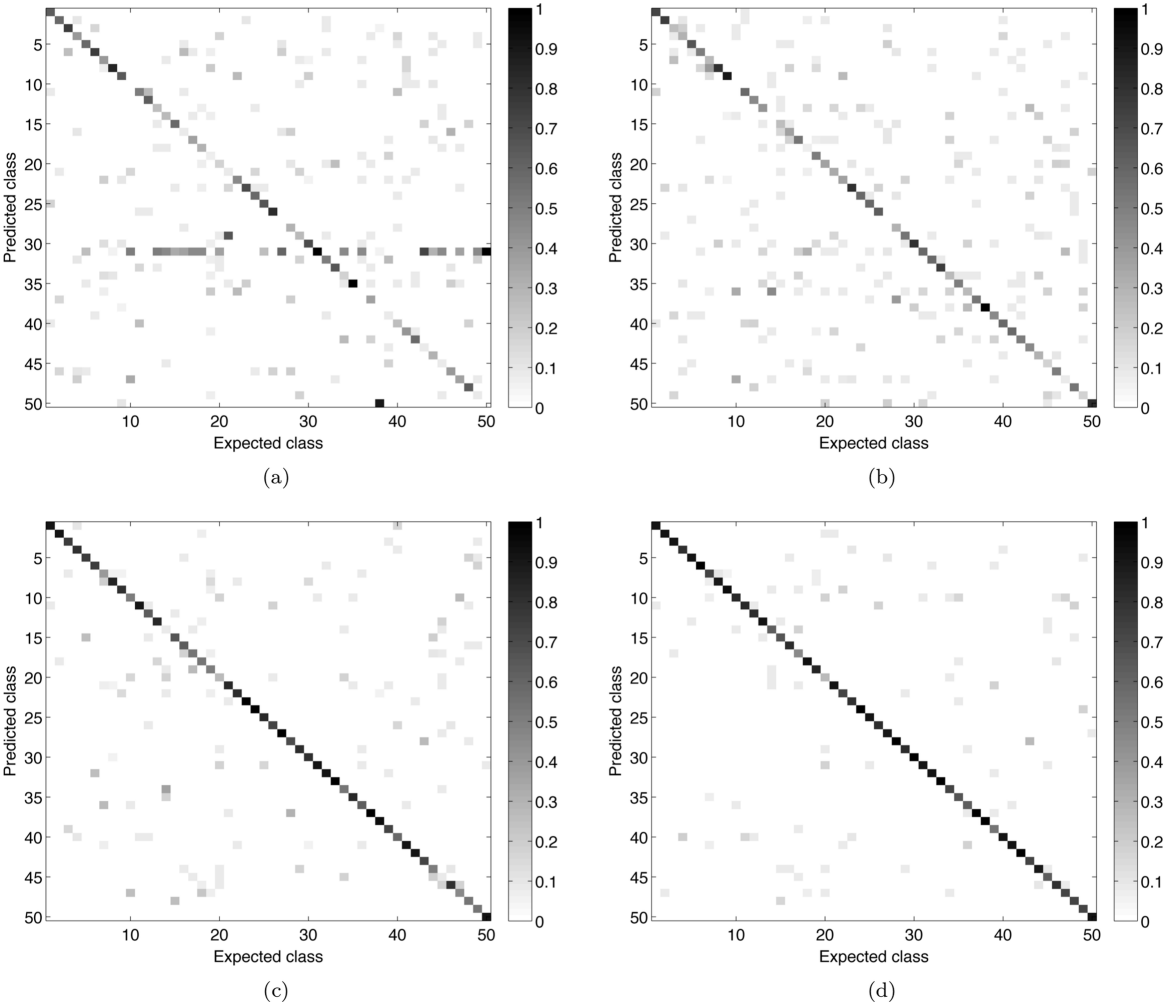
Confusion matrices of the main compared approaches. (a) Gabor descriptors. (b) Local Binary Pattern. (c) Bouligand-Minkowski fractal descriptors. (d) Combined fractal descriptors.

Fig 3 shows the confusion matrices for the main compared approaches (LBP, Gabor, Bouligand-Minkowski and the proposed method). Confirming its higher success rate value, the combined fractal descriptors provided the most accurate identification of the analyzed species. When compared to LBP and Gabor, the best performance of the proposed method is evidenced by the much smaller number of gray points outside the diagonal. When compared to Bouligand-Minkowski, the greater precision of the proposal is not so obvious, but it is observed for some classes, like 3 and 10. These are species where the cross-sections show more periodic patterns and where the frequency analysis gives relevant information.

**Fig 4:**
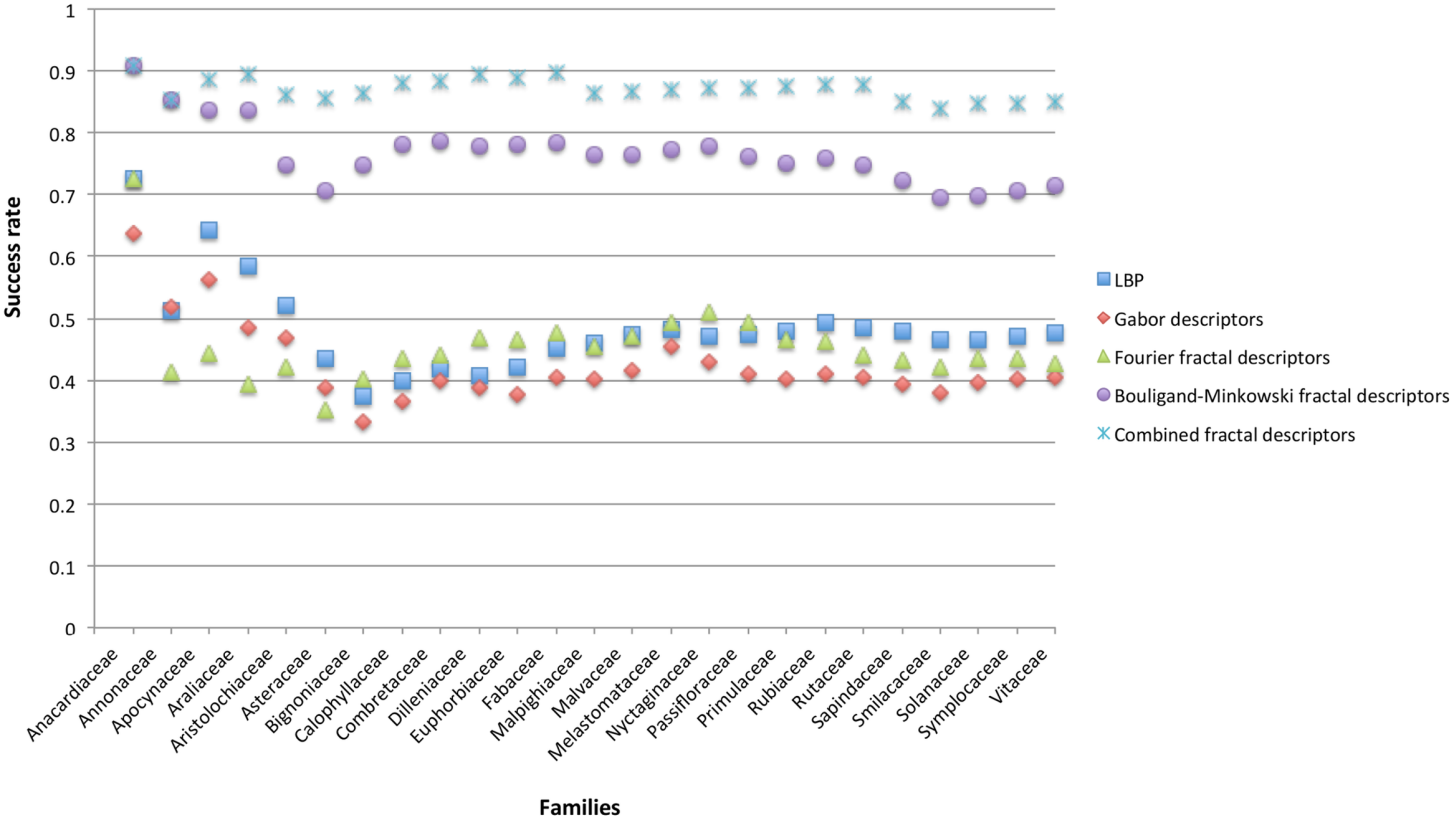
Averaged success rate of the species belonging to the same family considering the main compared approaches.

To verify how successful is the use of midrib in identifying species from the same family, the average success rate of the species belonging to the same family was calculated and presented at Fig 4. When the identification is performed considering the species, the proposed method achieved 83.67% of success rate, however, when the success rate of each family is calculated, the proposed method achieves 87.29% of correct identification. This means that at least 4% of the error is inside the family level, what is expected since the species belonging to the same family have substantial similarities.

The results above confirm what was expected from the theory background of each method concerning the perspective that each one shows from the image. Unlike Gabor, LBP and other approaches, fractal descriptors are conceived to model the natural composition law of biological structures. Such law is based on the self-replication of elements at different scales whereas this replication is also inherent to the self-similar nature of fractals. Particularly, the method proposed here combines two complementary ways of extracting fractal features. While the dilation volumes in Bouligand-Minkowski express the spatial morphology of the midrib, the Fourier method analyses the complexity of the frequency distribution. The combination by the KL transform results in a solution capable of identifying species using a simple and inexpensive setup and using a material that can be collected in most cases effortlessly at any time.

The identification of plant species using leaves is naturally a very challenging problem due to the high intra-species dissimilarity and inter-species similarity. Leaf variation occurs at every hierarchical level: within and among plants, populations, and species. In some species subject to different environmental conditions, marked phenotypic differences in leaves can occur during the development. Leaf variation within individuals may also occur regardless of environmental conditions, as part of the normal developmental pattern and seasonal changes, even among sequential leaf position on a stem. Nevertheless, the midrib proved to be a promising structure in the task of identifying plants.

In this context, the midrib of a leaf contains vascular bundles, associated fundamental tissues (parenchyma and/or collenchyma and/or sclerenchyma) and epidermis. Vascular tissues (xylem and phloem), which compose the midrib bundles vary in quantity and in their spatial disposal. In addition, the vascular system may be formed by a single bundle or be formed by a continuous or an interrupted arch, depending on species [19]. The characteristics of the fundamental tissues such as cell wall thickness, the presence of secretory cells or structures, and their distribution within the midrib also vary with the species. Similarly, depending on the species, the epidermis can vary depending on the presence or absence of trichomes and their type, the shape and size of its cells, cuticular thickness, etc [52]. Thus, anatomical studies that address the taxonomic aspect traditionally describe these tissues seeking some feature that can distinguish the species. The qualitative description of these features is a laborious task, however, quantitative data from midrib would be complicated to be obtained by methods which are commonly used in Botany. In this sense, the computational method proposed here obtained very informative measures of texture from the median ribs, being able to differentiate between species. For these reasons, this method is very promising for the present and forthcoming science, which has sought the automatic identification of species, facilitating studies across the wide diversity of plants occurring in the world.

## Conclusions

This study proposed to identify plant species of a tropical savanna of Brazil by extracting fractal descriptors of leaf midrib histological cross-sections. The proposed solution combines Bouligand-Minkowski and Fourier fractal descriptors to provide features for the leaf images. These features are categorized by a state-of-the-art classifier method, making possible the correct identification of the species.

The results confirmed what was expected from the fractal descriptors theory, thus the proposed method achieved a great precision in the species identification, outperforming other imaging techniques and making possible to obtain an automatic and precise categorization using basic biological procedures. We can also conclude that the midrib is a region of the leaf that can provide relevant information in the process of identification of plant species. Therefore, future studies should take into account both the characteristics of the median vein and of the mesophyll, which would increase the rate of discrimination among species.
